# Attachment security, verbal ability, and inhibitory control in middle childhood

**DOI:** 10.1186/s40359-021-00524-7

**Published:** 2021-02-04

**Authors:** Anna Kamza, Adam Putko

**Affiliations:** 1 Institute of Psychology, SWPS University of Social Sciences and Humanities, Chodakowska 19/31, 03-815 Warsaw, Poland; 2 Faculty of Psychology and Cognitive Science, Adam Mickiewicz University in Poznań, Poznań, Poland

**Keywords:** Attachment security, Cool and hot inhibitory control, Middle childhood, Verbal ability

## Abstract

**Background:**

The relationship between parent–child attachment and executive function (EF) in middle childhood remains relatively poorly studied. Very little is known about the role that the child’s verbal ability might play in these relationships. Therefore, in the present study, we explored the concurrent links between perceived attachment security with parents and hot and cool inhibitory control (IC)—a core component of EF—as well as the potential mediating role of verbal ability in those links.

**Methods:**

The participants were 160 children aged 8 to 12 (51% girls). They completed the Attachment Security Scale, the computerised version of the go/no-go task, the delay discounting task, and the vocabulary subtest from the Wechsler Intelligence Scale for Children. Pearson’s correlations were conducted to test relationships between the study variables. A hierarchical multiple linear regression analysis was performed to examine whether attachment security uniquely contributed to the outcomes after accounting for covariates. The indirect effects were tested using a non-parametric resampling bootstrap approach.

**Results:**

The results showed that, after accounting for the child’s age and sex, there was a direct relationship between attachment security with the father and cool, but not hot, IC. However, there were no significant links between attachment security with the mother and both aspects of IC. We also found that children’s verbal ability played a mediating role in the associations between both child–father and child–mother attachment security and hot, but not cool, IC above and beyond the child’s age.

**Conclusions:**

The current study extends previous work on executive functions in middle childhood. The results highlight the role of attachment in explaining individual differences in IC in middle childhood as well as the different mechanisms through which attachment with parents might explain cool vs. hot IC. The findings have potential implications for therapeutic interventions using the family context as a target to improve IC in middle childhood.

## Background

Inhibitory control (IC) is thought to be a core component of executive function (EF), a set of higher-order cognitive processes involved in the conscious control of thought, emotion, and action [1]. IC develops most intensively during pre-school age [2], but its further development also occurs in middle childhood and is believed to continue during adolescence [3]. Contributing to developmental changes and individual differences in a wide spectrum of cognitive and social outcomes (for a review, see [4]), IC (among the other components of EF) is found to be associated with numerous biological and socio-environmental factors. One of the significant variables in the latter group is the quality of the parent–child attachment relationship. Some studies of early childhood have shown that there are positive and direct links between children’s secure attachment to parents and their IC [e.g. 5, 6]. These links are explained mainly by pointing out that competent caregivers provide well-tuned external regulation [e.g. 7]. However, it cannot be precluded that the quality of attachment is associated with children’s IC not only directly but also through other factors. One factor might be the child’s verbal ability. The mediating role of this factor in the relationship between attachment and EF is suggested by results revealing a link between attachment and verbal ability [e.g. 8] and between verbal ability and EF [e.g. 9]. However, little is known about the links between those variables in middle childhood. Therefore, the purpose of our study was to examine whether there is a relationship between attachment and IC in children during this period of development and to what extent verbal ability mediates this relationship. In our study, we focus on IC as a central feature of the development of EF in childhood [e.g. 4] and one of the essential components of EF, and its cool and hot aspects. The development of the latter aspect occurs mostly in middle childhood, a relatively uninvestigated period when it comes to the relationship between attachment and IC.

### Executive function and inhibitory control

Despite some discrepancies regarding the way the construct of EF should be defined, most factor analyses indicate that it has a componential structure. Components of EF that have been identified include working memory, attentional flexibility, and inhibitory control [10]. Based on the results of neuroscience research, Zelazo and Müller [11] distinguished two different aspects of EF. The relatively “cool” control processes are associated primarily with the dorsolateral prefrontal cortex (DLPFC) and are evoked under relatively abstract, non-affective situations. On the other hand, the relatively “hot” top-down control processes are subsumed primarily by ventral and medial regions of the prefrontal cortex (VMPFC) and operate in motivationally and affectively significant situations. The existing evidence suggests that although cool and hot EF are related [1], their unique aspects differentially predict emotional and behavioural characteristics across childhood [e.g. 12].

One of the essential components of EF that emerges in all factor analyses of EF tasks is inhibitory control (IC). IC is defined as the ability to inhibit or suppress mental processes that are not relevant to the current goal or task [e.g. 10, 13]. IC is thought to be a key construct in the domain of self-regulation [14], as it underpins an extensive range of domains of functioning such as emotion regulation [e.g. 7], theory of mind [e.g. 15] and academic achievement [e.g. 16] across childhood and adolescence. Existing evidence suggests that in middle childhood, the maturation of hot IC is relatively more protracted and lags behind the development of cool IC [1].

From a neuroscience perspective, the development of EF is thought to be a result of the maturation of the prefrontal and orbitofrontal cortex [17, 18]. However, in the past decade, a growing interest in social factors related to individual differences in EF has emerged. Several variables pertaining to family background, including socioeconomic status (SES) [19], and mothers’ parenting practices, such as scaffolding [e.g. 20] or autonomy support [e.g. 21], have been implicated in EF development. Although there has been substantial research on predictors of EF in young children [for a meta-analysis, see 22], external factors relating to EF in older children have been explored far less. Moreover, research including both facets of EF—hot and cool—is still limited. Meanwhile, there are also notable developmental changes in EF during middle childhood that are likely to be influenced by the social environment [1].

It should be noted that IC also can be considered in the temperament-based framework, in which the term effortful control (EC) is used to describe the multidimensional construct consisting of “the efficiency of executive attention, including the ability to inhibit a dominant response, to activate a subdominant response, to plan, and to detect errors” [3, p. 129]. Therefore, the core function of EC is the use of attentional processes to regulate one’s emotional arousal, motivation, and behaviour. However, as some researchers claim [24–26], IC focuses primarily on top-down, volitional control of attention and cognitive self-regulatory processes (slower, relatively more effortful and deliberate), whereas EC includes primarily quick, automatic or nonconscious aspects of emotional reactivity and regulation. Therefore, in our study we adopt the distinction between hot and cool IC, which is rooted in the cognitive approach to self-regulation, and we consider IC from the cognitive perspective, as the core component of EF.

### Attachment and cognitive development

Child-parent bonds constitute the most intense and enduring relationships across childhood, and thus they are likely to be one of the prime candidates to account for environmentally-driven individual differences in children’s EF [5]. Child-parent attachment, as an important aspect of a caregiving relationship, is considered to be the main regulator of emotional, cognitive, and neurophysiological processes [27]. Therefore, the experience associated with this relationship is likely to be the basis for the self-regulating abilities in children.

From attachment theory, several hypotheses concerning the effects of child-parent attachment on the child’s cognitive development can be derived [28]. Given the objectives of the present study, one of these hypotheses seems to be of key importance. This is the social-network hypothesis [28], which predicts that secure children are more motivated to engage in frequent harmonious social interactions and to openly communicate with other people compared with insecure children. In a direct or indirect way (e.g. by contributing to the development of the child’s verbal ability), this stimulates cognitive development [28]. Moreover, parents of securely attached children tend to develop responsive and open communication with their offspring [29], and this was shown to engage the child in active construction of meaning, stimulate language development, and thus support the development of cognitive control [e.g. 30]. Given that in middle childhood parents still remain the child’s primary attachment figures [31] and considering the fact that it is a period of further significant growth in language skills (and a large part of children’s interactions with their parents is verbal), it seems reasonable to expect that language in this period is an important tool of EF development.

Interestingly, the role of child–father attachment in EF development is still mostly unexplored territory compared to child–mother attachment. On the other hand, a growing body of research suggests that attachment to the father is a significant factor in determining individual differences in specific domains of the child’s development [e.g. 32]. Moreover, some evidence suggests that there is a distinct change in importance of the father as a parent, especially concerning the support of exploration in middle childhood [33, 34]. According to some authors, fathers contribute to their children’s sense of security mostly by providing them with sensitive support during explorative and gently challenging play, whereas mothers contribute mainly by providing comfort when the child is in distress [35], and thus fathers are thought to play a more crucial role than mothers in the development of children’s exploration, autonomy and relationships with peers [33]. Some researchers claim that in the domain of cognitive activities, this specific paternal function results, for example, in higher levels of paternal than maternal demands in problem-solving tasks [36, 37, p. 992] and children’s academic performance [38]. Therefore, it seems that considering not only the child–mother but also the child–father attachment relationship is necessary to understand how attachment bonds with parents are organised in their potential influence on children’s EF development across childhood.

### Attachment as a predictor of executive function

Previous research on the links between children’s attachment and EF relates almost exclusively to early childhood. Bernier and colleagues [5] found that more securely attached children at 15 and 24 months of age showed a higher level of cool EF at 3 years of age, and this relationship remained significant even when the child’s language development, family SES, and maternal sensitivity were controlled. In a follow-up study, Bernier and colleagues [39] found that secure child–mother attachment at 2 years of age still remained a significant predictor of cool aspects of working memory, IC, cognitive flexibility, and planning ability in children 5‒6 years of age. The link between child–mother security and cool IC in pre-schoolers was also found by Heikamp and colleagues [40]. In turn, the longitudinal study by Low and Webster [41] showed that in comparison to secure attachment, disorganised attachment to the mother at 3 years was related to lower IC and planning ability at 6 years.

Regarding research on the links between attachment and hot EF, Mittal and colleagues [42] found that 2- and 3-year-olds with a secure or avoidant attachment to their mothers were able to control impulses more effectively than their ambivalently attached peers. Research has also shown that in comparison to children with disorganised attachment, secure children at 2 years of age achieved significantly higher scores at age 6 on a delayed gratification task [43]. Also, Moore and Symons [44] in their longitudinal study found that children who were securely attached to their mothers at age 3 were more likely to patiently wait for a prize at age 4 than their insecure peers. Finally, a positive relationship between secure attachment to the mother in children aged 6‒10 and the ability to delay gratification was also found by Marchetti and colleagues [6], the only study to date concerning middle childhood.

### Attachment as a predictor of verbal ability

Several authors suggested that the quality of parent–child relationships plays a formative role in the development of verbal ability, both in their receptive and expressive aspects [e.g. 45]. Although the association between attachment and verbal ability has been studied relatively rarely, existing studies show that indeed this link is quite strong (for a meta-analysis, see [28]). Child–mother attachment was found to be associated with language performance not only during infancy and pre-school ages [e.g. 46] but also during middle childhood and adolescence [e.g. 47]. However, in most of the existing studies of this type, child–father attachment is rarely included.

Meanwhile, some authors consider that fathers might play an even more significant role than mothers in children’s linguistic development, as they act as “a kind of linguistic bridge between the child’s familial environment and the outside world” [37, p. 999]. Existing research has shown that fathers placed higher language demands on their children (for a meta-analysis, see [48]). In fact, significant effects of child–father attachment security (measured observationally) on children's receptive language skills were found in infancy [49]. Moreover, self-reported attachment security with fathers was found to be the unique predictor of school children’s language mastery [37].

In explaining the associations between attachment and verbal ability, several hypotheses are relevant [28]. First, secure children might be more willing to communicate with their parents, and therefore they might be more exposed to adult language competence compared with insecure children [28]. Second, in secure dyads, parental responsiveness during teaching and conversations might enhance children’s communicative behaviour and linguistic ability. Furthermore, secure children have relatively more positive relationships outside the family, which in turn provokes a richer language environment.

On the other hand, Di Folco et al. [33] found that unlike attachment with the mother, narrative measures of attachment with the father were not related to the 6-year-old children’s verbal IQ. As those authors note, a possible explanation may be that compared to mothers, fathers use less supportive but more directive and informative language when interacting with their children. Hence, the socioemotional context evoked by the narrative measures probably elicits different linguistic skills, depicting these differences in the communicative context of the relationship to each parent [33, p. 728].

### Verbal ability and executive function

The role of language in EF is emphasised by both classical and contemporary theories of cognitive development [50]. Vygotsky [45] claimed that language is a necessary tool to verbally mediate one’s own behaviour and to solve problems. According to him, children learn vocabulary through verbal exchanges in social interactions, primarily with adults. Therefore, his theory provides the developmental mechanism for how social interactions might affect children’s higher-order cognitive processes through the mediating role of verbal ability.

Similar ideas are also present in more contemporary theories. According to Zelazo et al. [51], the development of cognitive control results from age-related increases in the ability to reflect on the rules children formulate and represent during problem-solving (see also [52]). In this context, language allows one to become aware of those rules. Also, labelling per se facilitates redirecting attention to crucial aspects of the task and thus directs self-reflection, which in turn promotes increased flexibility in thinking and acting [53]. All these claims are supported by numerous studies revealing significant links between children’s expressive or receptive verbal ability at varying ages and their performance on both cool and hot EF tasks [9, 53–55].

Although empirical research has begun to confirm the proposed importance of parent–child relationships in children’s EF development, the potential mediating role of verbal ability remains underexplored. To our knowledge, only one study has addressed this problem so far. In their longitudinal investigation, Matte-Gagné and Bernier [56] found that children’s expressive language mediated the relation between maternal autonomy support assessed in infancy and hot EF at the age of 3, above and beyond the child’s previous EF and SES. However, no such mediating role of language was found for cool EF. According to the authors, one explanation for these results is that maternal autonomy support had a direct influence on the development of cool EF. On the other hand, they claim that their hot EF task might be mediated by more simple forms of language compared with more complex cool EF task. Given the lack of replication studies, at the current state of research, it is not possible to rule out any of these potential explanations.

Of note, the mediating role of language in the relation between the children’s family environment and EF is also suggested by research focusing on aspects of that environment other than attachment bonds, such as parental behaviour and family SES. For example, Noble et al. [57] found in a sample of first-grade children that controlling for language ability eliminated the significant association between family SES and children’s cognitive control, raising the possibility that language mediates the link between those variables. In a study by Catale et al. [58], the relationship between parental educational status and children’s EF (aged 6–7 and 10–11) was partially mediated by children’s language skills. Recently, Lee et al. [59] showed that preschoolers’ language ability partially mediated the link between one aspect of parental scaffolding (adjustment of levels of support to create an optimal challenge for the child) and children’s EF.

## Summary

As our short review indicates, most of the current research on the links between child-parent attachment and EF is related to early childhood, ignoring the fact that EF continues to develop during middle childhood. Most of this research has focused on only one aspect of EF, but the question arises whether attachment security has the same consequences for EF in both cool and hot contexts. Finally, the weakness of previous studies is that they rarely consider the role of the father–child relationship. Meanwhile, as some authors indicate [e.g. 35], the relationship between the child and the father has slightly different characteristics than that with the mother. More specifically, mothers are preferred as a safe haven for soothing the child’s distress, while fathers are preferred as the secure base for exploration. The consequence of these differences might be distinct patterns of relationships between attachment to mothers and fathers and the two aspects of children’s EF.

Nevertheless, the mechanism through which attachment affects children’s EF has yet to be investigated. Evidence suggests that verbal skills are one of the most significant factors in EF development [e.g. 9]. Considering that verbal skills stem notably from parent–child interactions [45] and that child-parent attachment as the essence of a caregiving relationship is considered to be a significant factor in determining individual differences in child’s verbal ability [28], it seems reasonable to expect that there are indirect associations between child-parent attachment and children’s EF. Since our review of studies has shown that verbal ability and EF are to some extent related to family SES, we decided to control this variable in our study to see how specific the potential links between attachment, verbal ability, and EF are.

### The current study

Despite the growing interest in the role of child-parent attachment in EF development, little is known about how the attachment is related to individual differences in EF in middle childhood. Accordingly, the first purpose of this study was to examine the concurrent associations between attachment security with both parents and hot and cool EF in middle childhood (Objective 1). We also aimed to check the potential mediating role of the child’s verbal ability in those links (Objective 2). In our study, we focus on a crucial component of EF—inhibitory control and its cool and hot aspects. The development of the latter aspect occurs mostly in middle childhood—a relatively unknown period as far as the link between attachment and EF is concerned.

We expected that higher attachment security with parents would be related to the child’s better performance on hot and cool IC tasks (Hypothesis 1). We also predicted that those links would be mediated by the child’s verbal ability (Hypothesis 2). As the role of child–father attachment in children’s EF has not been researched much before, the potential differences in links between IC and child–mother vs. child–father attachment was an exploratory part of this study.

## Method

### Participants

The final sample consisted of 160 children aged 8 to 12 years (*M* = 123.29 months, *SD* = 16.53, range 96‒152 months; 51% girls). An additional five children took part in the study, but they were excluded from the analysis due to a large (> 25%) number of omission errors in the go/no-go task (one child), a lack of data due to the child interrupting this task (two children), or the loss of data due to a computer system error (two cases). Families lived in one of the large metropolitan areas in Poland, and their parents had higher (65% of mothers, 62% of fathers), secondary (30% of mothers, 32% of fathers), or basic education (5% of mothers, 6% of fathers).

### Materials and procedure

#### Attachment security

Children’s perceptions of attachment security were assessed using the Polish version [60] of the Attachment Security Scale [61], separately for child–mother and child–father relationships. The Attachment Security Scale is a self-report measure consisting of 15 items, which are rated using the Harter’s [62]) “Some kids…, but other kids…” format, and the children were asked to indicate which of four responses they would be most likely to make (“sort of like me” or ‘‘really like me”). The items tap the child’s belief in the responsiveness and availability of the attachment figure, the child’s use of the attachment figure as a safe haven, and the child’s reports of open communication with her or him. Scores across items were summed so that the higher scores on the Attachment Security Scale reflect greater attachment security with the given parent (range: 15‒60).

Previous research has demonstrated the validity of this measure [61, 63] with good internal consistency (for an overview, see [64]). The scale has significant associations with other attachment measures such as the ACSQ, Separation Anxiety Test (SAT), and caregiver sensitivity [64]. Furthermore, the Attachment Security Scale also showed significant associations with developmental correlates of attachment, such as school adaptation, emotional and peer social competence, self-esteem, and behavioural problems [63, 65]. The one-factor structure of the Polish version of the Attachment Security Scale was confirmed [60], and Cronbach’s alphas in the present study were 0.75 and 0.76 for security with mothers and fathers, respectively.

#### Cool IC

A computerised version of the go/no-go task [66] was used. In this task, the participant was required to respond to certain stimuli and to refrain from responding to others. The stimuli were the letters P and R. The participant saw on the computer screen an array (2 × 2) consisting of 4 squares, with one asterisk in each square. The letters were displayed serially in a random order in one of the four squares at the point where an asterisk was. The stimulus display time was 500 ms with an inter-stimulus interval of 1,500 ms. The task consisted of two parts, each involving 100 trials. In part 1, the target stimulus was the letter P, and the non-target stimulus was the letter R. In part 2, the response rule was reversed, so the participant was required to respond to the letter R and refrain from responding to the letter P. In each part of the task, the ratio of trials with the letter P to trials with the letter R was 80: 20. Before each part of the task, an instruction was displayed, which was also read aloud by the experimenter. The test trials in each part of the task were preceded by 10 practice trials. During the practice trials but not the test trials, participants received feedback on their reactions. The indicator of task performance was then the average proportion of no-go trials from both parts of the task in which the participant correctly refrained from responding (range 0‒100). The higher the score, the better the child’s inhibitory control. The duration of the go/no-go task was approximately 10 min, including task instructions and practice trials. The task reveals an acceptable test–retest reliability [67].

#### Hot IC

The delay discounting task, modelled on the temporal reward discounting procedure [68], was utilised as a measure of hot IC [1]. In this task, participants made choices between less valuable rewards (0.20, 0.40, 0.80, 1, 2, 3, 4, and 5 zlotys), which they could receive immediately, and a more valuable reward (6 zlotys), which they could get later (after 2, 5, or 7 days). For each delay, the large constant reward was paired with each of the small rewards and presented as a choice, e.g. “Would you prefer 1 zloty now, or 6 zlotys in 7 days?” Each small immediate reward was paired twice with every delay for the large reward, resulting in a total of 48 choice trials. To encourage participants to take their choices seriously, they were informed that after the completion of the task, one of their answers would be randomly drawn, and the amount of money indicated in the answer would be converted into a real prize (stationery item, mini-game, or key ring) and delivered to them after the time that was indicated in their response had elapsed. Based on the participant’s choices, for each of the three delay periods, an indifference point between two rewards—immediate and delayed—was estimated. To determine the indifference point for a given delay, the first trial always started with the test question about the participant’s preferences for the amount of 1 zloty available immediately and 6 zlotys available after a delay. Depending on the participant’s response, on the next trial, the amount of money offered immediately was decreased to the subsequent amount in the set (if the participant previously chose the immediate reward) or increased (if the participant already chose the delayed reward). Decreasing or increasing the amount of money was continued until a change in preferences, which meant that the indifference point, had been attained. Based on the indifference point data, the discount rate *k* was calculated for each delay period using the following formula: k = (A − V)/VD, where A = the value of the delayed reward, V = the value of the reward available immediately, and D = the delay [69]. The higher the discount rate, the stronger the tendency to lower the value of the reward over time, and the preference for the immediate reward. The dependent variable was the average value of discount rates for the three delay periods of 2, 5, and 7 days. To obtain an increasing index, the average discount rate was multiplied by − 1. Thus, the higher the value of this index, the better the child’s hot IC (range: − 16.58‒0). The task presents adequate test–retest reliability across a 1-week interval [70].

#### Verbal ability

The vocabulary subtest from the Wechsler Intelligence Scale for Children (WISC-R; [71]) in Polish (adaptation by Matczak and colleagues [72]) was applied. The child’s task in this test is to explain the meaning of a word given orally by the experimenter. The test measures verbal understanding, the ability to reproduce acquired knowledge, and the scope and adequacy of defining concepts. Estimates of split-half reliability for the Polish version of the Vocabulary subtest are similar in magnitude to its original version and range from 0.85 to 0.91 [72]. In the current study, due to time constraints, a version shortened to the first 20 items of the test was used. The indicator of verbal ability was the sum of points for answers (range 0‒40). The higher the score, the higher the level of the child’s verbal ability. The interrater reliability for the overall test score in the present study was high (Kendall’s W = 0.97; χ^2^ (24) = 89.96; *p* = 0.002).

### Procedure

After approval by the local ethics committee and permission from the local schools’ principals, invitation letters were sent to parents. They were invited to participate, provided that they had a child participant in the aforementioned age range who was living with both biological parents. Given also that EF is sensitive to neurological perturbations, such as, e.g. ADHD and learning disabilities [e.g. 68], children with those difficulties were excluded. The screening was based on the short sociodemographic questionnaire. Children were recruited with parental written informed consent; 1,000 families were asked, and 165 (17%) consented to their children’s participation.

Children were assessed individually by a female experimenter in a quiet room in the school they attended for two sessions about one week apart. In the first session, the following tasks were administered in a fixed^1^ order: go/no-go, and the Attachment Security Scale (child–mother version). In the second session the tasks were following: delay discounting task, Vocabulary test, and Attachment Security Scale (child–father version). The go/no-go task was presented using a laptop computer with a 15-inch screen, and the distance of the child from the screen was about 75 cm. The delay discounting task was presented in the paper-and-pencil form. The short demographic questionnaire was completed by the mothers of the children. As this study was part of a larger research project examining attachment, EF, and theory of mind, in addition to the above, several other tasks not related to the current study were administered. Upon completion of the study, children received small gifts (candy and stickers).

### Data preparation and analysis

Several preparation analyses were conducted on the go/no-go data to ensure reliable and valid estimates of the response parameters derived from this task. Similar to Bezdjian and colleagues [73], the accuracy and latency of responses were screened. Trials with response times shorter than 120 ms on the *n*th trial, combined with no reaction on the previous (*n* *− *1th) trial (R_*n*−1_ = no), were treated as missing and not taken into account in the analysis (*n* = 24). Children who did not respond to more than 25% of the Go trials (errors of omission) were also excluded from the analysis on account of having too few data points (*n* = 1).

Next, we analysed distributions of all our variables and screened them for extreme scores. No multivariate outliers were found, and the distributions of attachment security and the child’s verbal ability were within bounds of acceptable levels of skewness and kurtosis <  ± 1.00 [74] except for our cool (skewness = − 2.51; kurtosis = 8.72) and hot (skewness = − 5.76; kurtosis = 45.73) IC measures. Therefore, data from the go/no-go task were transformed by squaring the scores, which improved their parameters to the bounds of acceptable moderate normality (skewness = − 0.69; kurtosis = 0.68). Data from the delay discounting task were transformed by square rooting, which also improved their parameters (skewness = 0.54; kurtosis = 1.22). The transformed data were used in subsequent analyses.

All statistical analyses were performed using SPSS version 25. Descriptive statistics were calculated to summarise the data, and Pearson correlations were computed to compare relationships between the main variables with demographic variables as well as to determine their role as covariates.

A hierarchical multiple linear regression (MLR) analysis was performed to examine whether attachment security uniquely contributed to the outcome after accounting for covariates. MLRs were performed separately for the child–mother and child–father attachment. For each step, we report the increment in variance accounted for by the variables entered in that step, the standardised beta weights, and the squared semi-partial correlations (s*r*^2^).

According to the current approach to mediation analysis, which is related to the criticism of the classic Baron and Kenny [75] procedure [e.g. 76], a mediator variable can be intervening between predictor and outcome variables even if the latter two do not initially appear associated, as the total effect is a sum of many different paths, and some absent direct effect could result in significant indirect effects working in opposite directions [77]. Therefore, we tested the indirect effects using a non-parametric resampling bootstrap approach, as this method tends to have the highest power, the best Type I error control, and provides more accurate results than traditional tests with relatively small sample sizes [78]. We applied Model 4 of the PROCESS macro [79] with 20,000 resamples drawn with replacements from the original sample (*n* = 160) to derive the confidence interval (CI) for the unstandardised regression coefficient of the indirect effect. The indirect effect through verbal ability was considered significant when the CI did not include 0.

## Results

### Preliminary analyses

First, descriptive statistics and Pearson zero-order and partial correlations (controlling for age) for key study variables were calculated (see Table 1). We then examined the associations between both aspects of the child’s IC and demographic variables (child’s age, sex, and family SES), as well as the child’s performance on the verbal ability task.

**Table 1:** Zero-order correlations (below the diagonal), partial correlations (controlling for child age; above the diagonal) between the key study variables and descriptive statistics (N = 160)

	1	2	3	4	5	6	7	8
1. Sex^a^	–	–	.02	− .19*	− .07	− .03	.13	.05
2. Age^b^	− .03	–	–	–	–	–	–	–
3. Hot IC	.01	.18*	−	.18*	.07	− .00	.15	.26**
4. Cool IC	− .24**	.27**	.23**	–	.01	.17^#^	.06	.22**
5. M-security	− .03	− .05	.07	.03	–	.51***	.07	.18*
6. F-security	− .04	− .05	− .01	.14^#^	.49**	–	.02	.20*
7. SES^c^	.13	.10	.16^#^	.08	.07	.01	–	.28***
8. Verbal ability	.02	.53**	.27**	.27**	.14^#^	.16*	.29**	–
*M*	–	123.29	1.02	0.77	47.93	7.21	8.86	26.49
*SD*	–	16.53	1.71	0.13	6.47	5.25	2.39	6.07
Range	–	96–152	0.00–16.58	0.12–0.96	29–60	0–20	2.75–11.25	11–38

M-security = attachment security with mother; F-security = attachment security with father

^#^*p* < .1; *p < .05; **p < .01 (two-tailed)

^a^Child sex is coded: -1 = girl; 1 = boy

^b^Age in months

^c^*N* = 127 (due to the fact, that the SES questionnaires from 33 participants did not returned, and thus there was some missing data)

As displayed in Table 1 (below the diagonal), zero-order correlations revealed that cool and hot IC were positively and weakly related to each other. Furthermore, children’s verbal ability was positively linked to both hot and cool IC. Age was positively related to both aspects of IC and verbal ability. Sex (dummy coded) was related to cool IC only, with girls superior to boys. However, sex was not related to attachment security. Perceptions of attachment security to both parents were positively and moderately related. However, there were no significant differences in attachment security between child–mother and child–father attachment security, *F*(1, 159) = 1.20, *p* > 0.05, *η*_*p*_^2^ = 0.01. Finally, SES was positively related only to the child’s verbal ability.

After controlling for age (see Table 1 above the diagonal), almost all the relations mentioned above did not change substantially in their strength and direction. In light of these results, in the further regression analyses with cool IC as an outcome variable, age and sex were controlled. Since family SES was not significantly associated with the outcome variables, there was no reason to include it in the regression analyses. In turn, in the regression models with hot IC as an outcome variable, only age was covaried.

### Objective 1: links between attachment security with parents and IC

#### Cool IC

Table 1 presents Pearson's zero-order and partial correlations (controlling for age) between the child-parent attachment and both aspects of the IC. Regarding relations between attachment security with mothers and cool IC, we found no significant correlation between this variable and the child’s cool IC.

Regarding attachment to fathers, we found a weak association between attachment security and cool IC, which nevertheless did not approach significance, *r*(158) = 0.14, *p* = 0.07. However, since there was a significant correlation between cool IC, age, and sex that could suppress the correlation between IC and father–child attachment, we checked whether the latter variable would significantly predict cool IC when taking into account both age and sex. To this end, we ran a hierarchical linear regression analysis (Table 2). When the child’s age and sex were entered in the first step, they accounted for 11% of the variance in go/no-go task scores, *F*(2, 157) = 9.38, *p* < 0.001, with age and sex contributing uniquely (*p*s ≤ 0.01). After controlling for the covariates, attachment security with fathers became significant, β = 0.15, *p* < 0.05, and predicted an additional 2% of variance in cool IC, *F*(3, 156) = 3.77, *p* = 0.05. The entire model fit was good, *F*(3, 156) = 7.62, *p* < 0.001, and explained 13% of the cool IC variance. A similar analysis for mother–child attachment has shown that this variable remained a non-significant predictor of cool IC when both age and sex were taken into account, β = 0.04, *p* = 0.59; *F*(3, 156) = 7.15, *p* < 0.001.

**Table 2:** Hierarchical linear regression testing effects of perceived attachment security with father on child go/no-go task performance (N = 160)

Step	Inc. *R*^2^	*F*-change	β	*t*-value	s*r*^2^
Step 1	.11	9.38***			
Age			.23	3.02**	.23
Sex			− .23	− 3.08**	− .23
Step 2	.02	3.77*			
F-security			.15	1.94*	.14

The coefficients shown are those in the final model, while accounting for all other predictors. Child sex is coded: − 1 = girl; 1 = boy

*F-security* = perceived attachment security with father

*p* < .1; *p < .05; **p < .01; ***p < .001. Final model: *R*^2^ = .11; *F*(3, 156) = 7.57, *p* < .001

#### Hot IC

There were no significant correlations between child–mother or child–father attachment security and children’s hot IC even when age was taken into account (Table 1), so there was no basis for conducting a similar regression analysis for hot IC as an outcome.

### Objective 2: mediating role of verbal ability in the links between attachment security with parents and IC

In the next set of analyses, we tested whether children’s verbal ability served as a mediator in the links between attachment security and both aspects of IC (the hot and the cool). First, we examined associations between our potential predictors (i.e. attachment variables) and the potential mediator (i.e. verbal ability) and between the mediator and a given aspect of IC as an outcome [77]. In cases where these associations were significant, we examined a mediation model with attachment security as a predictor, children’s verbal ability as an intervening variable, and a given aspect of IC as an outcome using bias-corrected bootstrapping. Regarding the number of analyses (two) for each aspect of IC, we used α = 0.02 (following Bonferroni’s correction: 0.05/2 = 0.02) for all regression analyses of mediation.

Correlation analyses controlling for the child’s age (see Table 1 above the diagonal) revealed that security with both mothers and fathers was related to our potential mediator. In turn, children’s verbal ability was linked to both cool and hot IC. Recall that correlation analysis indicated that our attachment variables were associated with neither hot nor cool IC. However, considering that an indirect link can still be present despite a seemingly absent association between two variables [78], we investigated the indirect effects of our attachment variables on both aspects of IC.

#### Cool IC

For cool IC as an outcome variable, all mediation analyses were carried out with age as a control variable. Among the two mediation models tested, none of them were significant, suggesting that children’s verbal ability did not mediate the links between attachment security and cool IC (see Table 3).

**Table 3:** Mediation effect of verbal ability in the relationship between attachment security and cool/hot inhibitory control obtained in the bootstrap procedure

Model pathways	Cool IC^a^					Hot IC^b^				
	β	SE	*t*	*p*	95%CI	β	SE	*t*	*p*	95%CI
M-security										
a	.17	.06	2.57*	.01		.17	.06	2.55*	.01	
b	.21	.00	2.38	.03		.24	.01	2.57*	.01	
c	.04	.00	0.50	.62		.08	.00	1.01	.32	
c’	.00	.00			− .000; .095	.04	.00			.003; .098
F-security										
a	.19	.06	2.79*	.01		.18	.06	2.77*	.01	
b	.19	.00	2.07	.04		.26	.01	2.79*	.01	
c	.15	.00	1.94	.05		.00	.00	0.03	.98	
c’	.11	.00			− .004; .085	.05	.00			.008; .101

a—predictor relationship, b—mediator, c—direct effect, c’—indirect effect

*M-security* = attachment security with mother; *F-security* = attachment security with father

^a^Covariates: age and sex

^b^Covariate: age. β—standardised regression coefficient

^*^p ≤ .02 (following Bonferroni’s correction)

#### Hot IC

Regarding the two mediation models tested with hot IC as an outcome variable (controlling for age; see Table 3), the results suggest that attachment security, both with mothers and fathers, had an indirect effect on children’s IC. In both cases, full mediation occurred through children’s verbal ability.

### Supplementary analyses

To ensure the specifics of the results we had obtained, in supplementary analyses we explored whether perceptions of attachment security with parents have an integrative effect on IC. To this end, we averaged the security scores with both parents and then examined the effect of general perceived attachment security (*M* = 47.59, *SD* = 5.52; skewness = − 0.38, *SE* = 0.19; kurtosis = 0.37, *SE* = 38; range: 29–60) on IC. Hierarchical linear regression analyses revealed that the effect was significant neither for cool IC, β = 0.11, *p* = 0.15; *F*(3, 156) = 2.97, *p* = 0.05, controlling for child’s age and sex, nor for hot IC, β = 0.05, *p* = 0.54; *F*(2, 157) = 7.84, *p* < 0.001, controlling for child’s age, thus confirming our previous results that perceived attachment with the father had a unique effect on the child’s cool IC.

Analogically, to explore whether perceptions of attachment security with parents have an integrative effect on IC via verbal ability, in the additional analyses, we examined the indirect effect of general perceived attachment security on hot and cool IC through verbal ability. However, those effects were also not significant (for cool IC 95% CI = [− 0.001; 003]; for hot IC 95% CI = [− 0.107; 0.009]), indicating that the intervening effects were independent for attachment with the mother and the father as predictors.

## Discussion

The purpose of this study was to examine the concurrent links between children’s attachment security with parents and two aspects of IC, along with the intervening role of children’s verbal ability in those links. It was expected that higher attachment security with parents would be related to better hot and cool IC (Hypothesis 1). We also predicted that those links would be accounted for by the mediating role of children’s verbal ability (Hypothesis 2). The results only partially lend support for these hypotheses.

First, after accounting for the child’s age and sex, we found that there was a direct relationship between perceived attachment security with the father and cool IC. Namely, we observed that children with relatively higher attachment security with fathers had better cool IC. However, perceived security with the father did not directly predict hot IC. At the same time, there were no significant direct links between attachment security with the mother and both aspects of IC. Furthermore, our supplementary analysis also revealed that the effects of general perceived attachment security on cool and hot IC were not significant, confirming that perceived attachment with the father had a unique direct effect on the child’s cool IC.

Second, after accounting for the child’s sex, verbal ability mediated the links between attachment security with both parents and hot IC. Consistent with this, we found that children who were more secure with both parents had a higher verbal ability, explaining their greater hot IC. Furthermore, the indirect effects of general perceived attachment security on hot and cool IC through verbal ability were not significant, indicating that the observed intervening effects were independent for attachment with the mother and the father as predictors.

Our results seem to mirror the strong theoretical claims that the parent–child relationship plays a formative role in the child’s language and cognitive control [45]. However, the design of our study was correlational and cross-sectional; thus, longitudinal studies are needed to provide more reliable evidence suggesting a directional, and potentially causal, link between the attachment security and the child’s verbal ability and hot IC. Only such an approach would support the notion that secure attachment is associated with children’s verbal ability, thereby fostering their ability to inhibit impulsive responses [28, 29]. On the other hand, it is also worth noting that developing verbal skills per se might play a significant role in the process of forming and reconstructing working models of attachment [33], as the more advanced the child’s verbal ability, the better the ability to represent attachment relationships. This formative role of language is best reflected in attachment narrative measures, employing communication of emotions and needs and representational structure of memories [34]. Thus, the links between attachment and verbal ability may be bidirectional in their nature.

Our findings also seem to be in line with the well-established theoretical and empirical links between children’s verbal ability and cognitive control [e.g. 53] and especially with other research evidence that children’s receptive vocabulary and verbal self-instruction were related to the strategies used for waiting in delayed gratification measures [e.g. 80]. However, in the present study, we can only give post-hoc explanatory hypotheses for the mediational models found here. Given that the factors underlying the relationship between attachment and children’s verbal ability might be multiple and diverse (e.g. responsive and open communication of secure dyads or higher openness of secure children to social interactions) [28], the detailed mechanism of the impact of attachment on language development in middle childhood has yet to be investigated.

The findings also reveal that not only mothers but also fathers play a significant role in the emergence of individual differences in children’s language ability [e.g. 37] and in hot IC. Poor and superficial verbal exchanges during interactions with parents caused by their unavailability, unresponsiveness, or disapproval appear to undermine both the child’s verbal ability and impulse control.

In sharp contrast to the intervening effect of the child’s verbal ability on attachment security and hot IC, the analyses for cool IC suggest that children’s verbal ability did not mediate the relations between attachment security with parents and cool IC. The different patterns of links between attachment security and the two aspects of IC found in our study correspond to the findings of Matte-Gagné and Bernier [56]. They observed that children’s expressive language mediated the relation between maternal autonomy support in infancy and hot EF at the age of 3. In contrast, autonomy support was directly related to cool EF (unfortunately, paternal autonomy support was not included in this study). This finding seems to support the idea about the partial separateness of hot and cool IC (those two aspects of IC were positively but weakly correlated in our study) and the potentially different mechanisms underlying individual differences in hot and cool IC [56]. Regarding the fact that the maturation of hot IC lags behind the development of cool IC and that it develops rapidly in the transition to adolescence [1], one should consider the possibility that communication with parents might be more significant for the development of hot than cool IC in middle childhood. Another possibility might be that aspects of children’s language other than semantic are essential for cool IC in this developmental period.

Regarding the direct links between attachment to fathers and children’s cool IC, some evidence suggests that fathers’ involvement with their children increases as the offspring grow older (while mothers’ involvement is rather constant) [81], and that there is a distinct change in importance of the father as a parent, especially concerning the support of exploration in middle childhood [33]. Fathers are thought to play a more crucial role than mothers in the development of children’s exploration, autonomy, and relationships with peers, and some researchers claim that this specific paternal function results in higher levels of paternal than maternal demands in problem-solving tasks. Thus, one possible explanation for this finding might be that secure children have confidence in their fathers’ closeness and availability, which makes them feel freer to engage in an exploratory activity [27]. This kind of activity, in turn, might create for the child an opportunity to exercise their skills in planning and self-monitoring their actions and flexibly modifying them [28, 82]. Moreover, Grossmann et al. [35] propose that fathers contribute to children’s sense of security mainly by providing them sensitive support during explorative and gently challenging play. In contrast, mothers contribute primarily to providing comfort when a child is in distress. Hence, another possibility is that child–father interactions in middle childhood, especially those in the context of explorative and gently challenging play [35], might be particularly significant for shaping the child’s psychobiological mechanisms necessary to effectively control their behaviour and successfully solve the “cool” problems [83]. It is also possible that those specific patterns of links between security with the father and IC might be somewhat culture-specific. Lubiewska [84] pointed out that due to the fast cultural changes in Poland in the last decades, there exist micro-cultural discrepancies between relatedness-oriented mothers and their autonomy-oriented children. Therefore, as Polish fathers might be less overprotective and collectivistic in their socialisation goals than mothers, they might become primary attachment figures in the transition to adolescence. In turn, interactions with fathers might be more salient for child IC. However, longitudinal and culture-oriented explorations are needed to verify that hypothesis. It would be informative to also include some measures of both mother and father parenting and sensitivity in middle childhood for a more comprehensive comparison.

Finally, regarding the potential mechanisms including the role of verbal ability in the elaborated links, it cannot be excluded that more complex forms of language might mediate performance on our cool IC task compared with our hot IC task. This issue needs further investigation. Nevertheless, the fact that only attachment security with fathers (but not with mothers) predicted cool IC in middle childhood seems to suggest that the significant family contexts of IC development in middle childhood are the father–child interactions.

A large body of research reveals that SES is positively associated with language development [85], IC [86] and parental sensitivity [87]. The latter is thought to be one of the key feature of caregiver–child interactions, as it refers to the parent’s ability to notice the child’s signals, interpret them accurately and respond to them appropriately [88]. High maternal sensitivity has been proven to foster secure attachment in children, while lower maternal sensitivity has been linked to attachment insecurity [e.g. 63]. Maternal sensitivity is also associated with higher levels of language development and academic achievement [89, 90]. Life stress associated with low SES might result in household chaos, instability, parental depression, and more negative, punitive, and authoritarian parenting, which in turn leads to adverse developmental outcomes such as insecure attachment and disadvantages in language development [91]. As a result, one can argue that both SES and parental sensitivity may confound the results obtained in our study. However, as far as SES is concerned, we would like to note that SES in our study correlated weakly and insignificantly with attachment security with the mother (*r* = 0.07, *p* = 0.41) and with the father (*r* = 0.01, *p* = 0.83) as well as with cool (*r* = 0.08, *p* = 0.19) and hot (*r* = 0.16, *p* = 0.61) IC. SES was significantly correlated with verbal skills (*r* = 0.29, *p* = 0.001) only. This pattern of correlation precludes SES from explaining the relationship between attachment security and verbal abilities, as well as between verbal abilities and both cool and hot IC measures. Thus, the fact that SES was not controlled in the mediation analysis is justified by the fact that it did not significantly correlate with either the independent or dependent (outcome) variables. When it comes to parental sensitivity, we did not control for it in our study, so we cannot exclude its confounding role in our results. For example, attachment security might mediate the link between parental sensitivity and both the child’s verbal abilities and IC. Another possibility is that SES might moderate the links between attachment, verbal ability, and IC, with stronger links between those variables among children from low SES families. Future studies should control for both parental sensitivity and SES in order to exclude those other potential explanations.

### Limitations and future studies

In providing a novel link between attachment to both parents and hot and cool IC in middle childhood, the present study has some limitations. First, the magnitude of the significant correlations are quite small; however, they are comparable to some other studies, which are based on similar samples, fairly homogeneous in terms of the family environment, and utilising behavioural measures of EF [e.g. 39–41]. In those studies the magnitude of the significant links between attachment and EF was 0.08–0.31. Marchetti and colleagues [6] in their sample of school children also found a weak but significant link between attachment and hot IC (as measured with the delay discounting procedure, which was also used in our study), however this link became insignificant after accounting for the child’s age. On the other hand, there are some studies in which somewhat higher correlations between attachment and EF have been found [e.g. 5]. However, in most of those studies, composite scores of EF performance were analysed. Thus, one possible explanation is that these discrepancies might be due to attachment and EF measurement issues (see further discussion below). Another possibility is that the links between attachment and IC might weaken as children grow older so that some other (social) factors may contribute to individual differences in cognitive development. As far as the links between verbal ability and both age and hot IC are concerned, they are similar to those found, for example, by Carlson and Wang [7], with a moderate magnitude of relation between age and verbal ability (*r* = 0.54 vs *r* = 0.53 in our study), and quite comparable links between verbal ability and hot IC (*r* = 0.33 vs *r* = 0.27 in our study). Finally, links between the variables of interest might be moderated by SES, with stronger links in low SES families. Those alternative explanations should be explored in future studies.

We used a self-report measure to assess children’s attachment. There are some concerns about the validity of self-report measures, such as difficulties in conscious access to internal working models, the risk of response bias, and social desirability [e.g. 93]. Furthermore, using self-report measures of attachment to both parents could affect the results by shared method variance. Finally, it should be noted that attachment measures rely quite strongly on the child’s verbal skills [33, 34]. This fact raises the question of whether the observed link between attachment security and verbal ability is a function of the reliance on a questionnaire assessment [64]. Lab-based protocols, such as semi-projective story stems, observational codes, or attachment interviews may be more successful in assessing unconscious attachment processes. Therefore, following the recent suggestions [e.g. 93], future studies should adopt a multi-method approach to attachment measurement in order to corroborate the findings obtained.

Another limitation of our study is that the verbal ability index, based on the WISC vocabulary subtest, may reflect not only the level of verbal ability, but also of general intelligence. Therefore, it could be argued that what underlies the relationship between attachment measures and the Vocabulary subtest is not verbal ability but general intelligence. In fact, such an interpretation cannot be completely ruled out. However, some arguments speak against such an interpretation. First, as shown in the meta-analysis by van IJzendoorn et al. [28], the relationship between attachment and IQ is much weaker (*r* = 0.09) than that between attachment and linguistic competence (*r* = 0.28). Second, the Verbal IQ of the WISC-R correlates strongly (*r* = 0.70, *p* < 0.001) with the Linguistic Expression score of the CELF-R [e.g. 94]. Similar, and even stronger, correlations are noted between the WISC-R vocabulary subtest itself and the PPVT-R (e.g. *r* = 0.79, *p* < 0.001 [95]). This indicates that the WISC-R Verbal Scale, including the Vocabulary subtest, is strongly saturated with the language ability factor. This is in line with the opinion of Flanagan et al. [96] that the WISC vocabulary subtest poses strong language demands. Third, although it is widely believed that performance on EF tasks is related to general intelligence, research shows that such a relationship occurs primarily in the case of tasks measuring cold EF, in particular those that require updating working memory [cf. 97]. On the other hand, a weaker relationship is found between general intelligence and hot EF, including tasks measuring inhibition and set-shifting [97]. For example, in the only study known to us that used both the go/no-go and delay discounting tasks on a group of children aged 7 to 12 [98], weak and insignificant associations were found between full IQ and both of these tasks (*r* = − 0.05 in both cases), as opposed to the Digit Span task which correlated significantly (*r* = 0.22, *p* < 0.01) with the full IQ. Fourth, according to some theories of cognitive development [e.g. 51, 52], the growth in IC results from increasing capacities in the semantic aspect of verbal skills (i.e., verbal labelling, conceptual reasoning, and representation of the complex rules). And probably, that is why in most studies on EF, verbal skills were assessed with vocabulary tests. Nevertheless, other measures and other aspects of children’s language (e.g. self-directed speech or communicative competence) should be considered further to examine the links of interest. The data mentioned above make highly probable interpretation that not general intelligence but verbal ability underlies the relationship between the Vocabulary subtest and hot IC found in our study. To confirm this interpretation, future research should utilise a purer measure of linguistic ability than the WISC vocabulary subtest used in our study, and also control general intelligence, preferably with a non-verbal test.

Next, as we have already mentioned earlier—when a predictor, mediator, and outcome variables are measured simultaneously, other models would explain the data equally well (e.g. children’s verbal ability and IC might both influence the scores on attachment measure), and it is difficult to distinguish these alternatives without further investigation [77]. Furthermore, the study’s cross-sectional design did not allow us to examine whether there is developmental continuity or discontinuity in the patterns of the links between attachment to parents, verbal ability, and IC across middle childhood and other developmental periods. Therefore, systematic longitudinal research is needed to confirm the mediational links observed in our study and to clarify whether (and if so, how) those patterns of association change across different ages and which factors account for the potential change. Extending this investigation across and beyond the middle childhood years would also help to elucidate the causal processes underlying the development of individual differences in IC that are suggested by the current study.

Another limitation of the present study is its quite small sample size, which limits the generalisability of the results. In replication studies, more participants should be involved in each age group. Furthermore, the families were generally middle class, with most of the parents having a college degree. Hence, those results cannot be generalised to lower SES families. It is essential to replicate those results in more ethnically and economically diverse samples. Additionally, as we discussed above, family SES along with parental sensitivity may be confounding factors when studying the links between attachment, verbal ability and IC in children. Hence, in future studies not only SES but also parental sensitivity should be controlled.

Finally, given the well-documented low commonality of individual EF tasks [1, 12], it should be noted that the results of the present study might not be generalisable to other tasks measuring IC or to other EF components. Hence, future studies should include additional measures of IC and other components of EF, such as working memory and attentional flexibility. This approach would help us to answer whether the links observed in our study are EF-component-specific or more general.

## Conclusions

Taken together, our study was the first to investigate the links between attachment security both with mothers and fathers and the two aspects of IC in middle childhood. It adds to the existing body of research in several regards. First, it demonstrates the importance of child–father (in addition to child–mother) attachment in explaining both verbal ability and individual differences in IC in middle childhood. Second, our study suggests that there are different mechanisms in middle childhood through which attachment explains cool vs hot IC; namely, attachment security with the father is directly linked to cool IC in children, but there are no direct links between attachment security with the mother and IC. Verbal ability mediates the links between attachment security with parents and hot, but not cool, IC. Thus, our study provides some support for the notion that parent–child relationships may be an important factor contributing to individual differences in the child’s IC. More specifically, the results suggest that attachment security with the father may be more relevant for the development of cool IC than both attachment security with the mother and the child’s verbal ability. Besides, attachment with both parents seems to play a significant role in the child’s verbal functioning. The suggestion that the establishment of a secure parent–child relationship may be one of the mechanisms promoting the child’s cognitive development provides some encouragement to put strong emphasis on engaging both mothers and fathers in stimulating the development of children’s language and cognitive control. Regarding the well-documented capacity to improve the parent–child attachment relationship through some therapeutic interventions [e.g. 76], it seems reasonable to use the family context as a target of intervention to improve IC not only in younger children [5] but also in those in middle childhood.

## Data Availability

Data available from the corresponding author on request.

## Footnotes

1 Following Carlson and Moses' recommendation [99, p. 1035, footnote 1], given the correlational design of the study, we maintained the same order of methods for all participants.
